# Metabolic Reprogramming and Predominance of Solute Carrier Genes during Acquired Enzalutamide Resistance in Prostate Cancer

**DOI:** 10.3390/cells9122535

**Published:** 2020-11-24

**Authors:** Shiv Verma, Eswar Shankar, E. Ricky Chan, Sanjay Gupta

**Affiliations:** 1 Department of Urology, School of Medicine, Case Western Reserve University, Cleveland, OH 44106, USA; sxv304@case.edu (S.V.); exs334@case.edu (E.S.); 2 The Urology Institute, University Hospitals Cleveland Medical Center, Cleveland, OH 44106, USA; 3 Institute of Computational Biology, School of Medicine, Case Western Reserve University, Cleveland, OH 44106, USA; erc6@case.edu; 4 Department of Urology, Louis Stokes Cleveland Veterans Affairs Medical Center, Cleveland, OH 44106, USA; 5 Department of Nutrition, Case Western Reserve University, Cleveland, OH 44106, USA; 6 Division of General Medical Sciences, Case Comprehensive Cancer Center, Cleveland, OH 44106, USA

**Keywords:** enzalutamide resistance, castration resistant prostate cancer, metabolic reprogramming, solute carrier proteins

## Abstract

Androgen deprivation therapy (ADT) is standard-of-care for advanced-stage prostate cancer, and enzalutamide (Xtandi^®^, Astellas, Northbrook, IL, USA), a second generation antiandrogen, is prescribed in this clinical setting. The response to this medication is usually temporary with the rapid emergence of drug resistance. A better understanding of gene expression changes associated with enzalutamide resistance will facilitate circumventing this problem. We compared the transcriptomic profile of paired enzalutamide-sensitive and resistant LNCaP and C4-2B prostate cancer cells for identification of genes involved in drug resistance by performing an unbiased bioinformatics analysis and further validation. Next-Gen sequencing detected 9409 and 7757 genes differentially expressed in LNCaP and C4-2B cells, compared to their parental counterparts. A subset of differentially expressed genes were validated by qRT-PCR. Analysis by the i-pathway revealed membrane transporters including solute carrier proteins, ATP-binding cassette transporters, and drug metabolizing enzymes as the most prominent genes dysregulated in resistant cell lines. RNA-Seq data demonstrated predominance of solute carrier genes *SLC12A5*, *SLC25A17*, and *SLC27A6* during metabolic reprogramming and development of drug resistance. Upregulation of these genes were associated with higher uptake of lactic/citric acid and lower glucose intake in resistant cells. Our data suggest the predominance of solute carrier genes during metabolic reprogramming of prostate cancer cells in an androgen-deprived environment, thus signifying them as potentially attractive therapeutic targets.

## 1. Introduction

Prostate cancer is the second-leading cause of cancer-related mortality among men in the United States. Androgen receptor (AR) signaling is a critical survival pathway for prostate cancer cells, and androgen-deprivation therapy (ADT) through medical or surgical castration is the mainstay treatment for patients with locally advanced and metastatic disease [1]. Overall, the use of ADT has increased dramatically over recent years, with an estimated 500,000 men currently receiving ADT for prostate cancer alone in the United States [2]. The first generation antiandrogen, bicalutamide, fails to induce the precise conformational changes after binding to AR, resulting in limited efficacy [3,4]. This has led to the development of second generation antiandrogens that prevent AR translocation to the nucleus and inhibit downstream signaling or androgen biosynthesis [5]. Reports from several randomized phase III clinical trials have shown increased survival [6], particularly in patients with high tumor burden, when enzalutamide, a second generation antiandrogen, is started at the same time as initial ADT in a castration-sensitive environment [7]. Despite sustained response and initial benefit from enzalutamide, the majority of patients develop resistance and emergence of castration resistant prostate cancer (CRPC) [8].

Enzalutamide is a non-steroidal AR antagonist that binds the AR with higher affinity than conventional antiandrogens and impairs AR nuclear localization and transcriptional activity [9,10]. Enzalutamide received initial approval by the Food and Drug Administration (FDA) on August 2012, for the treatment of prostate cancer patients with hormone naïve or castration resistant tumors [11]. Enzalutamide competitively suppresses the binding of androgens to the AR, inhibits nuclear translocation and recruitment of cofactors, and inhibits binding of activated AR with DNA [12,13]. Moreover, recent studies have demonstrated that enzalutamide treatment significantly improves overall survival in CRPC patients [14,15]. Nonetheless, ~30% of these patients develop resistance over time, activating AR in these tumors [16,17]. Enzalutamide-resistant tumors represent a significant challenge because of a lack of other treatment options to prevent AR driven tumor progression but also due to rapid emergence of CRPC tumors. Unfortunately, serial specimens from post-enzalutamide treatment are difficult to obtain and may not reflect the full complexity of the disease.

Several mechanisms of adaptive resistance of prostate cancer to enzalutamide treatment have been identified. These aberrations include AR splice variants lacking ligand binding domain encompassing F876L mutations, imbalance in AR co-regulators, and bypass of AR in CRPC progression [18,19]. Unfortunately, efforts directed toward targeting some of these pathways in the clinical settings have not provided encouraging results. For example, recent studies have shown development of cross-resistance between enzalutamide with mTOR inhibitors and taxanes [20]. Such failures highlight the importance of systematic efforts toward discovering new mechanisms and pathways associated with the enzalutamide-resistant phenotype.

## 2. Materials and Methods

### 2.1. Cell Culture

Human prostate cancer LNCaP and C4-2B cells were used for the experiments. Cells were grown in RPMI 1640 (Cat# SH30027.01, GE Healthcare, Marlborough, MA, USA) supplemented with 10% fetal bovine serum, 50 U/mL penicillin, and 50 µg/mL streptomycin in 100-mm tissue culture plates at 37 °C in a humidified atmosphere (5% CO_2_). These cells were used for generating enzalutamide resistant cells and the absence of mycoplasma contamination was tested using a PCR-based assay (Cat# MP0025; Sigma-Aldrich, St. Louis, MO, USA).

### 2.2. Development of Enzalutamide Resistance

LNCaP and C4-2B cells were recurrently exposed to increasing concentrations of enzalutamide (1–20 μM) by passage in complete cell culture medium containing enzalutamide (Cat# A10562, Adooq Bioscience, Irvine, CA, USA). In each concentration of enzalutamide, the cells were grown in RPMI 1640 (GE Healthcare, Chicago, IL, USA) for a week, allowing them to acclimatize and propagate for a minimum of six months. The resistant cells generated from 20 µM enzalutamide were maintained in media containing 5 µM enzalutamide, referred to as LNCaP and C4-2B enzalutamide-resistant cells. The parental cell lines were propagated in dimethyl sulfoxide as the vehicle for the same time period. The final concentration of the vehicle did not exceed 0.1% in all treatments. Both cell lines were maintained in similar culture conditions.

### 2.3. RNA Isolation and NGS Sequencing

Total RNA was extracted from both LNCaP and C4-2B enzalutamide resistant and sensitive cells continuously exposed with enzalutamide (10 µM) using an RNA RNeasy Kit (Qiagen, Maryland, MD, USA). The total RNA integrity (RIN) was assessed using an RNA 6000 nanochip (Agilent Technologies, Santa Clara, CA, USA) on a Bio-analyzer 2100 (Agilent Technologies). Libraries were prepared using the Illumina TruSeq Stranded Total RNA Sample Preparation Kit according to the manufacturer’s protocol. The 50 bp single-end sequencing was performed on pooled libraries using an Illumina HiSeq 2500. Library preps and sequencing were completed by the Case Western Reserve University Genomics Core Facility.

### 2.4. RNA Sequencing and Data Analysis

Sequencing reads generated from the Illumina platform were assessed for quality using FastQC. Illumina HiSeq 2500 reads were trimmed and clipped for quality control in TrimGalore v0.4.3, a wrapper script for cutAdapt and FastQC. Alignment of the data was performed using STAR Aligner v2.5.3 using the human reference genome GRCh38 and the GENCODE transcript annotation v25. Differential expression was determined using Cufflinks v2.2.1. Differentially expressed genes were identified using a multiple testing corrected *p*-value < 0.05. Mitochondrial chromosome and the non-chromosomal sequences were excluded from the analysis. The Next-Gen sequencing data of LNCaP and C4-2B enzalutamide resistant cells were submitted to NCBI-GEO with accession numbers GSE150807 and GSE151083, respectively.

### 2.5. Quantitative Real-Time PCR

Total RNA was isolated from enzalutamide resistant and parental cell lines, and RNA quality was analyzed using a NanoDrop ND-1000 Spectrophotometer (NanoDrop, Wilmington, DE, USA). One µg total RNA was used for cDNA synthesis (Applied Biosystems™, Foster City, CA, USA) using a High-Capacity cDNA Reverse Transcription Kit (Thermofisher, Waltham, MA, USA). To quantify and amplify the gene oligonucleotides designed by Integrated DNA Technologies (Coralville, IA, USA) were used. The list of the genes probed are mentioned in Supplementary Materials Table S1, and GAPDH (NM_008084), and Actin (NM_007393) were used as the internal controls in the reaction. All reactions were performed in triplicate (three biological and three technical replicates) along with no template controls (NTC). The reaction for qRT was set up accordingly: 2.5 µL of SYBR green (Radiant™ SYBR Green low-ROX qPCR, Alkali Scientific, Fort Lauderdale, FL, USA) of 5× sample were added for a total 10 μL volume with thermal cycler program used started at 50 °C for 2 min then proceeded with 95 °C for 10 min for initial denaturing, followed by 40 cycles of 95 °C for 15 s, 60 °C for 40 s, and 72 °C for 35 s to collect cycle threshold (Ct) values, along with the dissociation curve cycle. The 2^−ΔΔCT^ method was used to calculate the relative expression of each gene as previously described [21].

### 2.6. Pathway and Gene Set Enrichment Analysis

The pathway analysis was performed using iPathwayGuide (Advaita, Ann Arbor, MI, USA). The signaling pathways were categorized and ranked on the basis of the number of target molecules overlapped in a particular pathway. Analysis was conducted with the upregulated and downregulated genes greater than or equal to 2-fold change or less than or equal to 2-fold change, respectively. The *p*-values indicate the significance of enrichment for the most highly expressed genes from the dataset. *p*-values were corrected for false discovery rate (FDR) using Benjamini-Hochberg (B-H) test. Gene set enrichment analysis of significantly differentially expressed genes in LNCaP and C4-2B cells was performed using GSEA V3.0 and MSigDB (Broad Institute, Cambridge, MA, USA) and cross referenced to the Gene Set Enrichment Analysis (GSEA), REACTOME, Kyoto Encyclopedia of Genes and Genomes (KEGG), and Gene Ontology (GO) datasets.

### 2.7. Gene Network Analysis

The gene network analysis was performed using Network Analyst, a visual analytics platform for comprehensive gene expression profiling and meta-analysis. Transcription factor and gene interaction was analyzed using human annotation. The transcription factor target database inferred from integrating literature curated Chip-X data was used to generate the gene network.

### 2.8. Lactic Acid Assay

The Amplite™ Colorimetric L-Lactate Assay Kit (Cat# 13815; AAT Bioquest, Sunnyvale, CA, USA) was used to perform the lactic acid assay. The reaction was incubated at room temperature for 2 h, protected from light, and the absorbance ratio increase was monitored with an absorbance plate reader at A575 nm/A605 nm. The data were analyzed as per the vendor’s instructions.

### 2.9. Citric Acid Assay

The MitoCheck^®^ Citrate Synthase Activity Assay Kit (Cat# 701040; Cayman, Ann Arbor, MI, USA) was used to perform the experiment and followed the instructions as per the manufacturer’s directions.

### 2.10. Glucose Uptake Assay

Glucose uptake assay was performed using a Glucose Uptake-GloTM Assay Kit (Cat# J1341, Promega, Madison, WI, USA). Briefly, 2DG uptake was initiated by the addition of labeled 2-deoxy-D [1–3H] glucose to a final concentration of 1 mM per well (1Ci = 37 GBq). Incubation was performed for 10 min at room temperature. For standardization, cells were counted using a Neubauer hemocytometer chamber before each experiment. A total of 2.5 × 105 LNCaP parental cells and enzalutamide resistant cells were employed in each assay.

### 2.11. Western Blotting

The parental LNCaP and enzalutamide resistant cells, grown in 100 mm dishes, were lysed in high-salt buffer containing 50 mmol/L HEPES (pH 7.9), 250 mmol/L NaCl, 1 mmol/L EDTA, 1% NP-40, 1 mmol/L phenylmethylsulfonylfluoride (PMSF), 1 mmol/L sodium vanadate, 1 mmol/L NaF, and protease inhibitor cocktail (Cat# 11836153001; Roche, St. Louis, MO, USA). The cell lysate was cleared by centrifugation at 13,000 rpm for 15 min at 4 °C, and the protein concentration was measured in the supernatant by the Bio-Rad assay following the vendor’s protocol (Bio-Rad Laboratories, Hercules, CA, USA). A total of 35 µg of protein was resolved by sodium-dodecyl sulfate–polyacrylamide gel electrophoresis (SDS–PAGE) using 4–20% Tris-glycine polyacrylamide gel and then transferred onto the nitrocellulose membrane overnight. The blots were blocked using 5% nonfat dry milk in Tris-buffered saline containing 0.05% Tween-20 and probed using primary antibodies incubated overnight with anti-AR (Cat# 5153), anti-AR-v7 (Cat# 68492S) and anti-PSA (Cat#2475S) from Cell Signaling Technologies, Danvers, MA, USA. Anti-SLC12A5 (Cat# A15486) and anti-SLC25A17 antibodies (Cat# A14840) were purchased from Abclone, Woburn, MA, USA, whereas SLC27A6 (Cat# 7297) was purchased from ProSci, Poway, CA, USA. The anti-GAPDH (Cat# sc-365062) antibody used as a loading control was procured from Santa Cruz Biotech, Dallas, TX, USA. Following secondary antibody incubation, immunoreactive proteins were visualized with an enhanced chemiluminescence detection system (Cat# XR94, BrightStar, Alkali Scientific, Fort Lauderdale, FL, USA).

### 2.12. Statistical Analysis

The significance between the enzalutamide resistant cells and the parental controls were performed by the Student’s *t*-test and *p* values less than 0.05 were considered as significant. The qRT-PCR data were analyzed using the two tailed unpaired *t*-test.

## 3. Results

### 3.1. Development of Enzalutamide Resistant Prostate Cancer Cell Lines

Human prostate cancer LNCaP and C4-2B cells were used to generate enzalutamide resistant cells. Androgen-responsive LNCaP cells were derived from a needle biopsy taken from the left supraclavicular lymph node metastasis and harbor point mutation at AR containing the threonine to alanine mutation of amino acid 877. This mutation has been observed in both naïve and castration-resistant patients [22]. The C4-2 subline is a derivative of LNCaP cells grown in castrated nude mice. Finally, the C4-2B cell line was developed from a bone metastasis by orthotopic transplantation of C4-2 cells in athymic nude mice [22]. The LNCaP and C4-2B tumor models mimic advance-stage, poorly tumorigenic, androgen-responsive, and non-metastatic prostate cancer in LNCaP to metastatic and androgen-refractory/castration-resistant in C4-2B tumors, which are excellent in vitro models for androgen deprivation therapy. Both cell lines were treated continuously with enzalutamide and the cell fraction that survived was pooled, maintained, and compared with their parental counterparts. Phenotypically, enzalutamide resistant cells were heterogeneous in nature, having loss of cell-to-cell tight contact with scattered growth and temporary arm-like projections. The growth curves for both cell lines demonstrated a marked difference in their sensitivity to enzalutamide treatment. These changes were clearly correlated with the loss of AR and AR-v7 and decrease in the expression of downstream effector molecule PSA in enzalutamide resistant cells (Supplementary Figure S1A–C).

### 3.2. Comparison and Validation of Gene Expression Levels between Enzalutamide Resistant and Parental Cells

**Figure 1:**
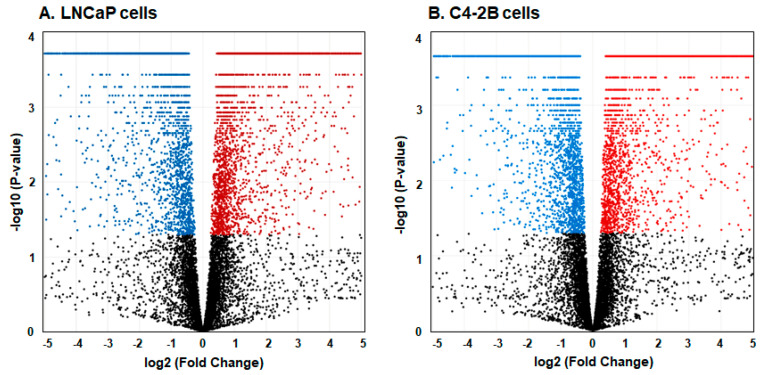
Volcano plot of (**A**) LNCaP enzalutamide resistant and (**B**) C4-2B enzalutamide resistant cells compared to their parental counterparts. The volcano plot displays the relationship between magnitude of gene expression change (log2 fold-change; X-axis) and statistical significance of this change [−1og10 of false discovery rate (FDR); Y-axis]. Each dot represents an individual gene. Red dots indicate the upregulated genes with Log2 fold-change > 2 p adjusted (FDR) <0.005 and blue indicate downregulated genes with Log2 fold-change > 1.2 p adjusted (FDR) <0.01, while the black dots represents non-significant genes.

Data analysis of 35,504 expressed genes, 9409 genes were differentially expressed (DEGs) were identified in LNCaP enzalutamide resistant cells (NCBI-GEO accession# GSE150807). In C4-2B enzalutamide resistant cells, 33,027 expressed genes and 7757 DEGs were identified (NCBI-GEO accession# GSE151083). Among those 9409 DEGs in LNCaP enzalutamide resistant cells, 4578 transcripts were upregulated and 4184 were downregulated; whereas 7757 DEGs in C4-2B cells, in enzalutamide resistant cells, 3728 transcripts were upregulated, and 3960 were downregulated at *p* value < 0.0005 and FDR< 0.05. To further visualize the DEGs, a volcano plot was generated displaying the relationship between the magnitude of gene expression change (log2 fold-change; X-axis) and statistical significance of this change [−log10 *p*-value after Benjamini-Hochberg adjustment; Y-axis] in comparison between LNCaP and C4-2B enzalutamide resistant and sensitive cells (Figure 1A,B).

**Figure 2:**
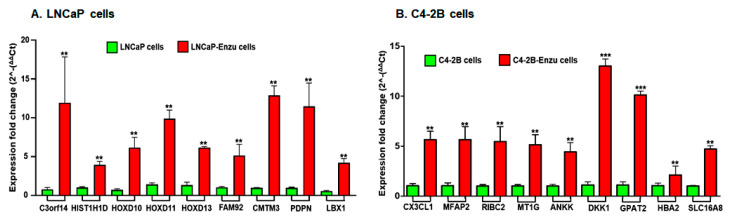
Real time PCR validation of genes in (**A**) LNCaP cells. Bars represent mRNA expression analysis of *C3orf14*, *HIST1H1D*, *HOXD10*, *HOXD11*, *HOXD13*, *FAM92*, *CMTM3*, *PDPN*, and *LBX1* genes differentially expressed between LNCaP enzalutamide-resistant cells compared to the parental cell line. (**B**) C4-2B cells. Bars represent mRNA expression analysis of *CX3CL1*, *MFAP2*, *RIBC2*, *MT1G*, *ANKK*, *DKK1*, *GPAT2*, *HBA2*, and *SLC16A8* genes differentially expressed between C4-2B enzalutamide-resistant cells compared to the parental cell line. The qRT-PCR data were analyzed using REST© (Relative Expression Software Tool), Qiagen, USA. Bar represents the standard error mean (SEM) for three biological and three technical replicates. ** *p* < 0.001, *** *p* < 0.0001 Control versus enzalutamide resistant cells.

In order to validate our findings obtained from the RNA-Seq data, we selected a subset of the top ranked nine genes that were differentially upregulated in LNCaP and C4-2B enzalutamide resistant cells with a log2 fold change, compared to the parental cell line. These genes were evaluated by qRT-PCR. The fold change gene expression of these genes in LNCaP cells including *C3orf14*, *HIST1H1D*, *HOXD10*, *HOXD11*, *HOXD13*, *FAM92*, *CMTM3*, *PDPN*, and *LBX1* were in agreement with the expression of the RNA-Seq data. Higher gene expression of *C4orf14* (13.9 fold), followed by *CMTM3* (13.8), *PDPN* (12.8), *HOXD11* (9.8), *HOXD10* (6.17) *HOXD13* (6.17), *FAM92* (5.16), *LBX1* (4.12), and *HISD1H1D* (3.94) in their expression were noted in the enzalutamide resistant cells compared to the LNCaP parental cells (Figure 2A). The fold change gene expression of these genes in C4-2B cells including *CX3CL1*, *MFAP2*, *RIBC2*, *MT1G*, *ANKK*, *DKK1*, *GPAT2*, *HBA2*, and SLC16A8 were in agreement with the expression of RNA-Seq data. Higher gene expression of *DKK1* (13.0 fold), followed by *GPAT2* (10.12), *CX3CL1* (5.65), *MFAP2* (5.6), *RIBC2* (5.4), *MT1G* (5.15), *SLC16A8* (4.77), *ANKK* (4.43), and *HBA2* (2.12) in their expression were noted in C4-2B enzalutamide resistant cells compared to the parental cells (Figure 2B).

### 3.3. Pathway Enrichment Analysis and Mining of Disease Association

**Figure 3:**
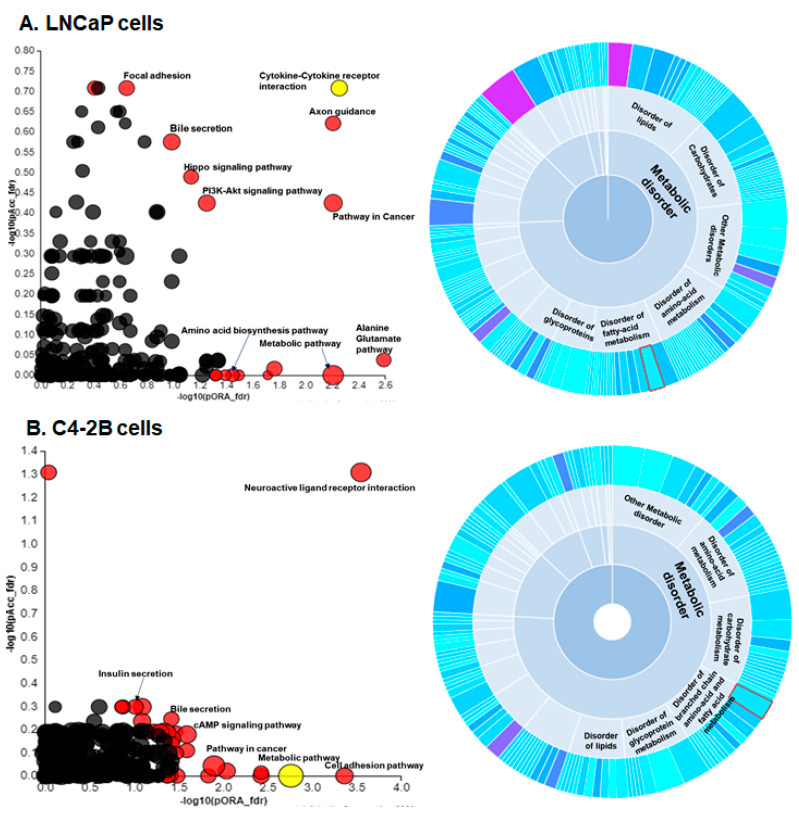
Pathway enrichment analysis of disease association in (**A**) LNCaP enzalutamide resistant cells and (**B**) C4-2B enzalutamide resistant cells compared to their parental counterparts. Overrepresented signaling pathways were analyzed by iPathway. Red color dots represent the pathway after FDR correction and yellow dots represent top hit pathways such as cytokine-cytokine receptor interaction in LNCaP cells and metabolic pathway in C4-2B cells. The colored dots denote the overrepresented pathways with corrected *p* value (FDR < 0.05) (Left panel). The circular plot displays significantly enriched pathways associated with the disease. Both LNCaP and C4-2B enzalutamide resistant cells exhibited DEGs linked with metabolic disorder. The circular plot of metabolic disease represent the DEGs genes overlaid with International Classification of Diseases, Tenth Revision (ICD-10). The metabolic disease display color magenta is the most significant and cyan is less significant (right panel).

We next performed signaling pathway analysis using iPathway on differentially expressed genes to investigate their biological relevance and pathway association. To achieve this, the data were separately analyzed with upregulated (fold change > 2) and downregulated (fold change < −2) DEGs. Analysis of iPathway showed overrepresented pathways associated with DEGs that included focal adhesion, bile secretion, Hippo signaling, PI3K-Akt signaling, cytokine-cytokine receptor interaction, axon guidance, pathways in cancer, amino acid biosynthesis pathway, metabolic pathway, and alanine glutamate pathway in LNCaP cells (Figure 3A). In C4-2B cells, DEGs include neuroactive ligand receptor interaction, insulin and bile secretion, cAMP signaling, and cell adhesion pathways (Figure 3B). Signaling pathway associated with cellular metabolism including alterations in amino acid, bile acid biosynthesis, salts, and glucose were noted to be commonly overrepresented in both LNCaP and C4-2B enzalutamide resistant cell lines compared to their parental counterparts.

Next, we analyzed the DEGs and their disease association through the circular plot. The plot displays significantly enriched pathways associated with the disease. Both LNCaP and C4-2B enzalutamide resistant cells exhibited DEGs that were linked mainly with metabolic disorder (Figure 3A,B, right panel). Further clustering showed disease association with disorder of lipids, carbohydrates, fatty acid, and metabolism of branched chain amino-acids, fatty acids, and glycoproteins (Figure 3A,B, right panel). The data showed that the ratio of the number of genes associated with the metabolic disorder was significantly high compared to other diseases when corrected using FDR.

### 3.4. Gene Set Enrichment Analysis (GSEA)

**Figure 4:**
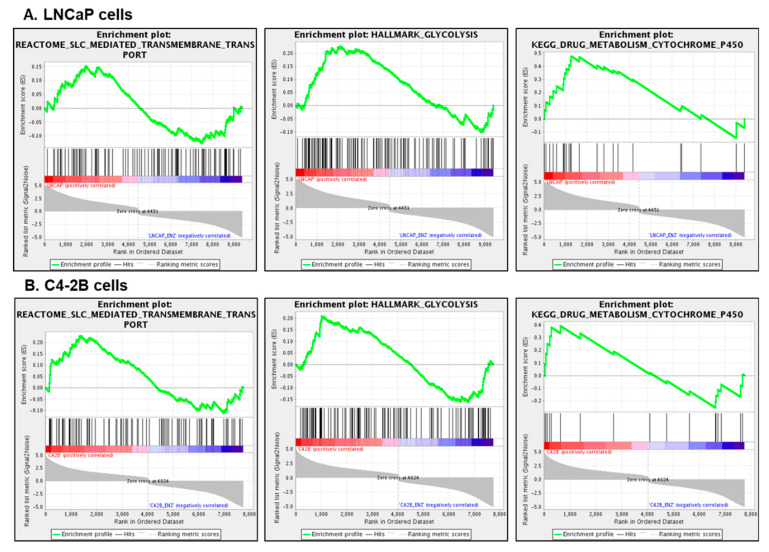
Gene set enrichment analysis. (**A**) LNCaP enzalutamide resistant cells and (**B**) C4-2B enzalutamide resistant cells, compared to their parental counterparts at a threshold of FDR *p*-value < 0.05. Enriched pathway includes SLC-mediated transmembrane transport, glycolysis, and drug metabolism. The green curve corresponds to the enrichment score (ES), which is the running sum of the weighted enrichment score obtained from GSEA software. Red and blue color bar display the positive and negative regulated genes.

We analyzed the DEGs with gene set enrichment analysis (GSEA) v3.0 (http://software.broadinstitute.org/gsea/downloads.jsp) to identify genes, their expression, and correlation associated with metabolic pathway between LNCaP and C4-2B enzalutamide resistant cells with their parental counterparts. The GSEA plots from REACTOME, Hallmark, and KEGG demonstrated significant gene enrichment and overrepresentation of metabolic pathways in both cell lines including SLC-mediated transmembrane transport, glycolysis, and drug metabolism at a threshold of FDR *p*-value < 0.05 (Figure 4A,B).

**Figure 5:**
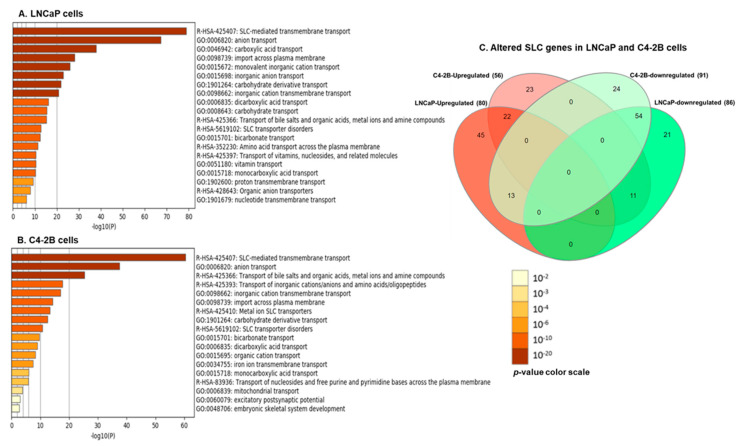
SLC transporter gene enrichment analysis. (**A**) LNCaP enzalutamide resistant cells (**B**) C4-2B enzalutamide resistant cells, compared to their parental counterparts at a threshold of FDR *p*-value < 0.05. The bar color of enrichment analysis of DEGs is displayed in both cell lines with *p*-values. (**C**) Venn diagram exhibiting altered SLC genes in both cell lines. The red zone represents upregulated SLCs and the green zone exhibits downregulated SLCs.

Next, enrichment analysis of SLC genes was performed in both cell lines. The data identified SLC associated signaling pathways such as SLC mediated transmembrane transport, anion transport, carbohydrate transport, transport of bile salt, transport of inorganic cations/anions, and amino acids/oligopeptides and other membrane transporters to be significantly upregulated in LNCaP and C4-2B enzalutamide resistant cells compared to the parental counterparts (Figure 5A,B). The Venn diagram represents common upregulated or downregulated genes in both LNCaP and C4-2B enzalutamide resistant cells (Figure 5C). The red zone represents upregulated SLCs and the green zone exhibited downregulated SLCs. The analysis identified 80 upregulated and 86 downregulated SLCs in LNCaP cells; whereas 56 upregulated and 91 downregulated SLC genes were noted in the C4-2B cell line. Among the upregulated SLC genes, 45 and 23 SLCs were particularly expressed in thee LNCaP and C4-2B enzalutamide resistant cells, respectively, whereas 22 SLCs were commonly expressed in both cell lines. Moreover, 21 and 24 SLCs were particularly downregulated in the LNCaP and C4-2B enzalutamide resistant cell lines, while 54 were common among these downregulated SLC genes in both cell lines (Supplementary Materials Table S2).

**Figure 6:**
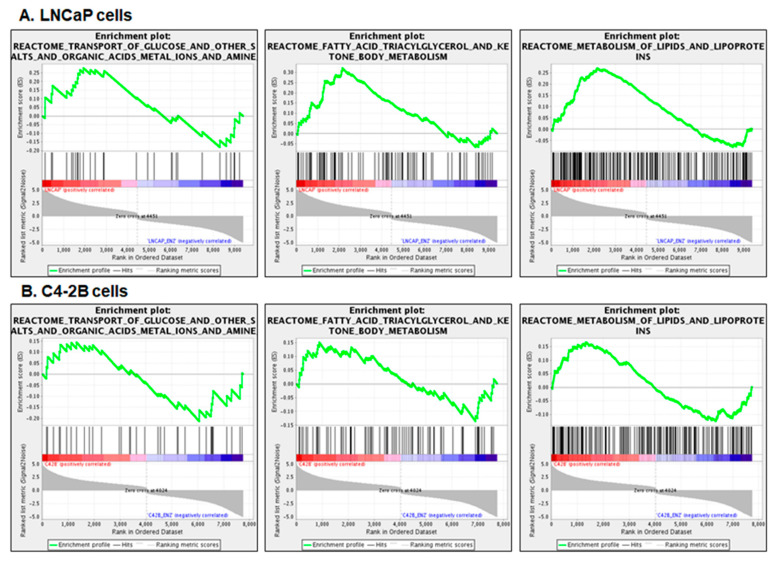
Gene set enrichment analysis. (**A**) LNCaP enzalutamide resistant cells and (**B**) C4-2B enzalutamide resistant cells compared to their parental counterparts at a threshold of FDR *p*-value < 0.05. Enriched pathway includes fatty acid metabolism, glucose transport, and metabolism of lipid and lipoproteins. The green curve corresponds to the enrichment score (ES), which is the running sum of the weighted enrichment score obtained from GSEA software. Red and blue color bar display the positive and negative regulated genes.

Next, we analyzed the transcriptomic data of LNCaP and C4-2B enzalutamide resistant cell lines aligned with GSEA analysis. The results exhibited overrepresentation of cellular metabolic pathways as a result of increased glycolysis and drug metabolism transport in the enzalutamide resistant cells compared to their parental counterparts. The resistant cell lines demonstrated changes in the transport of glucose, amines, amino acids, and fatty acids across cellular membranes. The GSEA plots from REACTOME demonstrated that significant gene enrichment of the fatty acid metabolism, glucose, and metabolism of lipid and lipoproteins altered at a threshold of FDR *p*-value < 0.05 (Figure 6A,B). Similar results were obtained from GO and KEGG database analysis (data not shown). Since both cell lines exhibited metabolic disorder and overrepresentation of SLC genes common in enzalutamide resistant cells, further studies were conducted in the LNCaP enzalutamide resistant and parental counterpart.

### 3.5. Identification of Genes Dysregulated in Glucose, Fatty Acid, and Lipid Metabolism

**Figure 7:**
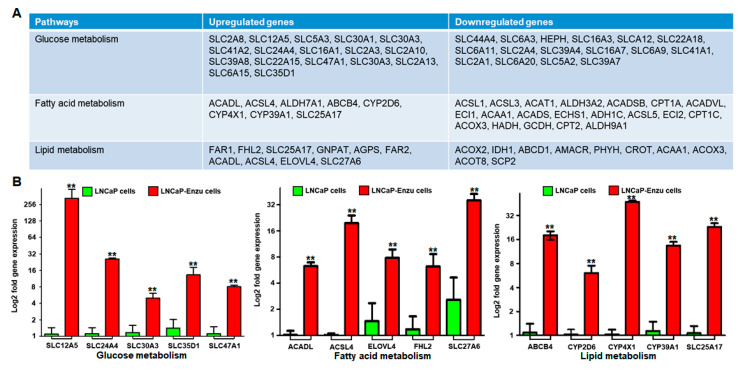
(**A**) Alteration in gene expression associated with the regulation of glucose, fatty acid, and lipid metabolism in LNCaP (parental) and LNCaP enzalutamide resistant cells. (**B**) Real time PCR validation of genes of glucose metabolism *SLC12A5*, *SLC24A4*, *SLC30A3*, *SLC35D1*, and *SLC47A1*; fatty acid metabolism *ABCB4*, *CYPD6*, *CYP4X1*, *CYP39A1*, and *SLC25A17*; and lipid metabolism *ACADL*, *ACSL4*, *ELOVL4*, *FHL2*, and *SLC27A6*. The qRT-PCR data were analyzed between LNCaP versus LNCaP-enzalutamide resistant cells using (delta-delta CT method; fold = 2^−ΔΔCT^). Bar represents the standard error mean (SEM) for three biologicals and three technical replicates. ** *p* < 0.001, LNCaP versus LNCaP enzalutamide resistant cells.

Next, the subset of genes altered in glucose, fatty acid, and lipid metabolism in LNCaP versus LNCaP enzalutamide resistant cells were analyzed (Figure 7A). In glucose metabolism, several solute carrier (SLC) genes are involved in the transport of glucose in cells. Among the SLCs, the expression of *SLC12A5* was markedly higher (1195 fold) in enzalutamide-resistant cells, while *SLC24A4* showed 20-fold higher expression, followed by *SLC35D1* (9.5), *SLC13A4* (5.0), *SLC30A3* (4.6), and *SLC47A1* (4.3) fold change in LNCaP enzalutamide resistant cells compared to the parental cells (Figure 7B).

Genes associated with fatty acid metabolism including *ACADL*, *ACSL4*, *ELOVL4*, *FHL2*, and *SLC27A6* were analyzed and validated. Among them, expression of *SLC27A6* showed 17.3 fold increased expression followed by *ACSL4* (15.8), *ACADL* (14.7), *FHL2* (6.0), and *ELOVL4* (3.0) gene expression fold change high in enzalutamide resistant cells compared with their parental counterparts (Figure 7B).

Finally, genes associated with lipid metabolism viz. *ABCB4*, *CYP2D6*, *CYP4X1*, *CYP39A1*, and *SLC25A17* were analyzed and quantified. The expression level of CYP4X1 was highest (30.0), followed by SLC25A17 (17.5), ABCB4 (15.0), CYP39A1 (8.5) and CYP2D6 (6.7) fold change high in enzalutamide resistant cells compared with the parental counterparts (Figure 7B).

### 3.6. Altered Expression of Glycolysis End Product and SLC Protein Expression

**Figure 8:**
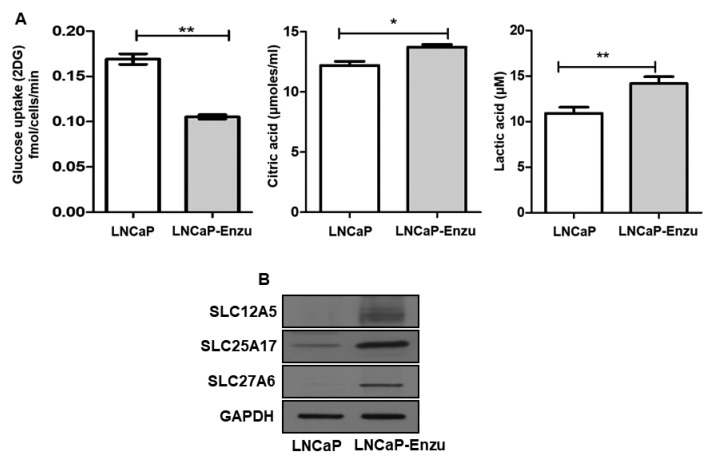
(**A**) Glycolytic end product metabolic uptake assay performed in LNCaP (parental) and LNCaP enzalutamide resistant cells. Glycolysis end products viz. glucose uptake, citric acid, and lactic acid were measured. LNCaP enzalutamide cells showed elevated level of citric acid (*p* value = 0.05) and lactic acid (*p* value = 0.01); whereas glucose uptake was higher in LNCaP cells compared to LNCaP enzalutamide resistant cells (*p* value = −0.01). Bar represents the standard error mean (SEM) for three biological and three technical replicates. * *p* < 0.05, ** *p* < 0.001, LNCaP versus LNCaP enzalutamide resistant cells. (**B**) Western blotting for protein expression of SLC12A5, SLC25A17, SLC27A6 in LNCaP, and LNCaP enzalutamide resistant cells. GAPDH expression as a loading control.

Next, we performed metabolic assays in LNCaP enzalutamide resistant and parental cell lines. The results demonstrated a marginal increase in citric acid (13.7 µmoles/mL) in LNCaP enzalutamide resistant cells compared to parental cells (12.2 µmoles/mL) at *p* < 0.05 while overproduction of lactic acid was observed in LNCaP enzalutamide resistant cells (14.17 µM) compared to LNCaP cells 10.9 µM at *p* < 0.05. The glucose uptake revealed higher uptake in LNCaP cells compared to LNCaP enzalutamide resistant cells (Figure 8A). We also confirmed the expression of SLC25A17, SLC12A5, and SLC27A6 proteins by western blotting, which were higher in the LNCaP enzalutamide-resistant cells compared to the parental counterparts (Figure 8B).

### 3.7. Solute Carrier Genes and Network Analysis

**Figure 9:**
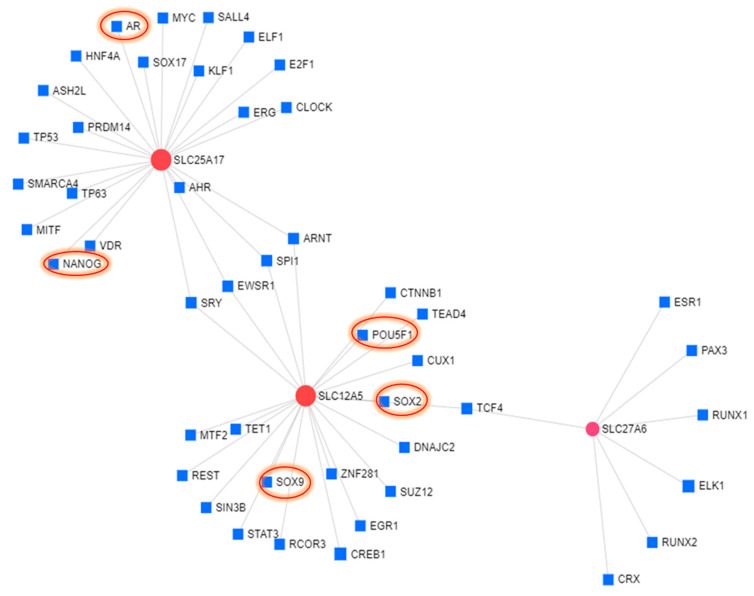
Gene network analysis performed using Network Analyst. Transcription factor targets database inferred from integrating literature curated Chip-X data was used to generate the gene network.

The gene network built demonstrated three SLCs genes including *SLC25A17*, *SLC12A5*, and *SLC27A6* as key regulators of metabolic reprogramming in LNCaP cells. Among the above three SLCs, *SLC25A17* gene network analysis showed its interaction with AR, TP53 TP63, SMARCA4, DLF1, E2F1, NANOG, SOC17, and others while SLC12A5 showed interaction with SOX2, POU5F1, SOX9, STAT3, REST, EGR1, SUZ12, and others. SLC27A6 showed interaction with RUNX1, ESR1, RUNX2, and others (Figure 9).

## 4. Discussion

The clinical use of the second-generation antiandrogen drug enzalutamide has improved the quality of life and survival of patients with castration-resistant prostate cancer. Unfortunately, approximately 30% of patients develop tumor resistance against enzalutamide over a period of 18–24 months treatment [16], and this remains one of the major challenges in the management of advanced-stage prostate cancer. The molecular pathways and the target molecules responsible for the emergence of drug resistance have not been fully discovered. In the present study, we identified a potential mechanism of resistance to enzalutamide. Our data demonstrate the predominance of membrane transporters including solute carrier genes (SLCs), ATP-binding cassette transporters, and other drug metabolizing enzymes as direct targets during metabolic reprogramming after prolonged enzalutamide treatment. Our data, for the first time, show that the involvement of solute carrier genes (SLCs) in the dysregulation of glucose, fatty acid, and lipid metabolism pathways during androgen deprivation promote enzalutamide resistance.

Altogether, in the present study, 9409 and 7757 DEGs were identified in LNCaP and C4-2B enzalutamide resistant cells. In LNCaP cells, upregulated gene expression of *C3orf14*, *HIST1H1D*, *HOXD10*, *HOXD11*, *HOXD13*, *FAM92*, *CMTM3*, *PDPN*, and *LBX1* were noted. In C4-2B cells, *CX3CL1*, *MFAP2*, *RIBC2*, *MT1G*, *ANKK*, *DKK1*, *GPAT2*, *HBA2*, and *SLC16A8* were identified as upregulated genes in enzalutamide resistant cells. These findings were validated by real-time PCR. Analysis of canonical pathways revealed metabolic reprograming as the hallmark of enzalutamide resistance in prostate cancer overrepresented by increased fatty acid metabolism, drug resistance signaling, drug metabolism, glucose and bile acid biosynthesis, lipid metabolism, and disorder of carbohydrates and glycoproteins in both cell lines. These results were further confirmed by gene set enrichment analysis. Previous reports on metabolomics profiling on men undergoing androgen deprivation therapy identified increased metabolites associated with bile acid, fatty acid, and type II diabetes [23].

In this study, the most pronounced altered pathways correlated with the enrichment of glucose, fatty acid, lipid metabolism, and bile acid biosynthesis associated with metabolic reprogramming of tumor cells. Genes regulating glucose metabolism in LNCaP enzalutamide resistant cells including several solute carrier proteins such as SLC2A8, SLC12A5, SLC5A3, SLC30A1, SLC30A3, SLC41A2, SLC24A4, SLC16A1, SLC2A3, SLC2A10, SLC39A8, SLC22A15, SLC47A1, SLC30A3, SLC2A13, SLC6A15, SLC35D1, and others were upregulated; whereas SLC44A4, SLC6A3, HEPH, SLC16A3, SLCA12, SLC22A18, SLC6A11, SLC2A4, SLC39A4, SLC16A7, SLC6A9, SLC41A1, SLC2A1, SLC6A20, SLC5A2, SLC39A7, and others were downregulated. Alteration of these genes have been demonstrated to be involved in the regulation of glucose metabolism and facilitate enzalutamide resistance. In particular, high expression of the above-mentioned genes were documented previously in relationship to drug resistance and conferring sensitivity to anticancer agents [24,25,26].

In our study, genes regulating fatty acid and lipid metabolism in LNCaP enzalutamide resistant cells including *ACADL*, *ACSL4*, *ALDH7A1*, *ABCB4*, *GNPAT*, *AGPS*, *FAR2*, half LIM-domain protein 2 (*FHL2*), elongation of very long chain fatty acids (*ELOVL*)-2 and 4 (*ELOVL2*, and *ELOVL4*), *SLC25A17* and *SLC27A6* were upregulated. Genes belonging to the ATP-binding cassette (ABC) transporter family, acetyl-coenzyme, aldehyde dehydrogenase, and synthesis of bile acids and bile salts including *ACSL1*, *ACSL3*, *ACAT1*, *ALDH3A2*, *ACADSB*, *CPT1A*, *ACADVL*, *ECI1*, *ACAA1*, *ACADS*, *ECHS1*, *ADH1C*, *ACSL5*, *ECI2*, *CPT1C*, *ACOX3*, *HADH*, *GCDH*, *CPT2*, *ALDH9A1*, *ACOX2*, *IDH1*, *ABCD1*, *AMACR*, *PHYH*, *CROT*, *ACAA1*, *ACOX3*, *ACOT8*, and *SCP2* were downregulated. ACADL is involved in catalyzing the final reaction of triglyceride synthesis and its overexpression is frequently observed in patients with obesity and type 2 diabetes [27,28]. Increased abundance of ACSL4, SLC27A6, ELOVL2, and ELOVL4 are involved in anticancer drug resistance, and their aberrant expression leads to metabolism and restoration of lipids and fats, essential for maintenance of cell proliferation [29,30]. High expression of SLC25A17 is involved in sterol synthesis, and its mechanistic regulation has yet to be elucidated. The ATP-binding cassette transporters such as ABCB4 is responsible for translocation of several substrates across cell membranes including steroids, phospholipids, and glycolipids [31]. High expression of ABCB4 has previously been documented in context with anticancer drug resistance either by modification of drug targets and genetic reprogramming of cellular metabolites to bypass target pathways [32,33]. Expression of ACSL4 was particularly increased in castration-resistant prostate cancer, compared with hormone naive prostate cancer [34,35]. Our study also demonstrated upregulation of several cytochrome P450 genes (CYPs) viz. *CYP2D6*, *CYP4X1*, and *CYP39A1* in enzalutamide resistant cells. CYPs are involved in drug metabolism and synthesis of cholesterol, steroids, and other lipids. High expression or activity of CYPs lead to drug resistance and poor therapeutic outcomes.

Solute carrier genes (SLCs), a family of more than 300 members comprising of 52 families in humans, facilitate the transport of a wide array of substrates across biological membranes. SLCs play a key role in physiological processes ranging from the cellular uptake of nutrients to the absorption of drugs and other xenobiotics. Our results indicate that predominance of SLC genes in context with enzalutamide resistance is not cell line-specific and indeed linked with castrate resistant prostate cancer. The prominent changes in the expression of SLCs regulate metabolic demands of resistant cells for sugar, amino acids, amine, nucleotide, and other metabolites. Furthermore, in-depth analysis of RNA sequencing data demonstrates the predominance of the SLC gene family. In our study, aberrant expression of *SLC12A5*, *SLC25A17*, and *SLC27A6* were strongly implicated in metabolic reprogramming and development of chemotherapeutic drug resistance. Solute carrier family 12 member 5 (SLC12A5) is a potassium chloride cotransporter 2 that has been identified as an integral membrane KCl cotransporter that maintains chloride homeostasis. Studies suggest that overexpression of SLC12A5 has a pivotal oncogenic role in carcinogenesis through the inhibition of apoptosis and promoting metastasis by regulating key elements of the matrix architecture [36]. The SLC25 gene encodes mitochondrial carriers such as membrane-integrated proteins that localize to the inner membranes of mitochondria and catalyze the translocation of solutes across the membranes [36]. These mitochondrial carriers provide a critical link between the mitochondria and the cytosol by facilitating the flux of solutes through the permeable barrier of the inner mitochondrial membrane. The SLC27 gene family members are involved in the transport of long-chain fatty acids frequently associated with the regulation of cell behaviors including cancer cells. Upregulation of SLC12A5, SLC25A17, and SLC27A6 both at the transcript and protein level, in the present study, were associated with an increased uptake of lactic and citric acid and a corresponding decrease in glucose uptake in enzalutamide resistant cells compared to their parental counterparts. Considering the fact that interaction of these three SLC genes with their respective downstream or upstream regulators together is associated with the development of drug resistance, we further analyzed the TF-gene interactions of three SLCs (SLC12A5, SLC25A17, and SLC27A6) using Chip-X data, and the results revealed that SLCs promote stemness in enzalutamide resistant cells. SLC12A5 displayed interaction with SOX2, SOX9, and POU5F1 (OCT4), whereas SLC25A17 exhibited interaction with stem cell transcription factor, NANOG. It will be interesting to understand the potential association between SLCs and transcription factors regulating stemness in enzalutamide resistant cells.

## 5. Conclusions

Taken together, our data strongly indicate that solute carrier genes play major roles in metabolic reprogramming of prostate cancer cells in an androgen deprived environment, resulting in progression and recurrence of castrate-resistant prostate cancer. Consequently, they are potentially attractive therapeutic targets.

## Supplementary Materials

The following are available online at https://www.mdpi.com/2073-4409/9/12/2535/s1, Figure S1: Effect of enzalutamide treatment to LNCaP and C4-2B cells, Table S1: List of primers, Table S2. List of SLCs-genes differentially expressed in LNCaP and C4-2B-enzalutamide resistant cells.
