# Supplementary material for: Metabolic Reprogramming and Predominance of Solute Carrier Genes during Acquired Enzalutamide Resistance in Prostate Cancer
Source: Cells. 2020 Nov 24;9(12):2535. doi: 10.3390/cells9122535 (PMC7759897; doi:10.3390/cells9122535)
Supplement: Supplementary file 1 [file cells-09-02535-s001.pdf]

Figure S1

Effect of enzalutamide treatment to LNCaP and C4-2B cells.

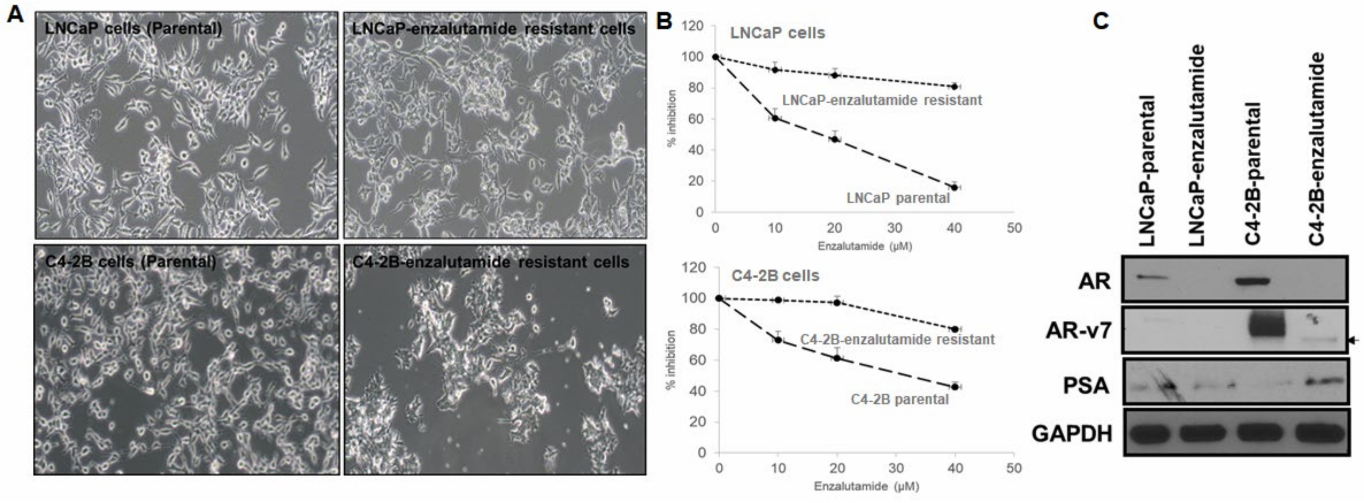

Figure S1. (**A**) Phenotype changes in LNCaP and C4-2B cells after enzalutamide exposure. Microscopic picture of LNCaP and C4-2B parental and enzalutamide resistant cells at 10x magnification. (**B**) Sensitivity to enzalutamide treatment in LNCaP and C4-2B parental and enzalutamide resistant cells. The cells were treated with increasing doses of enzalutamide (10-40 µM) in 0.1 % DMSO for 48 h. Cell viability was measured by MTT assay. (**C**) Protein expression of androgen receptor (AR), AR-v7, and prostate specific antigen (PSA) in LNCaP and C4-2B enzalutamide resistant cells and their parental counterparts by Western blotting. GAPDH as loading control.

Table S1. List of primers

Symbol	Genes	Sequence (5' to 3')
ABCB4	ABCB4_For_L_Enzu	TGTCTCAGGAGCCTATCCTATT
	ABCB4_Rev_L_Enzu	GGCTGCACTCACAATTTCATC
ACADL	ACADL_For_L_Enzu	GCCCTACTTGGAGAAGAGAATAAA
	ACADL_Rev_L_Enzu	CACTAGCTGAAATTGCCACATC
ACSL4	ACSL4_For_L_Enzu	ATATCGCTCTGTCACACACTTC
	ACSL4_Rev_L_Enzu	GCTGTCCTTCTTCCCAAACT
ANKRD26P1	ANKRD26P1_For_qRT_C42B-Enzu	TTCGACAGCAACTGGATGAG
	ANKRD26P1_Rev_qRT_C42B-Enzu	GCTCTTCACTCTGAGCTTGAT
C3orf14	C3orf14-qRT-lnCap-Enzu_For	TGGGTGATCAACACACAGAAA
	C3orf14-qRT-lnCap-Enzu_Rev	ATCTCTAGCAGCAAGCACTG
CMTM3	CMTM3-qRT-lnCap-Enzu_For	CAGGCGTCTAAACAGCAAAG
	CMTM3-qRT-lnCap-Enzu_Rev	GCCAAGAAAGTCGCAAGAAA
CYP26B1	CYP26B1_For_L_Enzu	GCGTCATCAAGGAGGTCAT
	CYP26B1_Rev_L_Enzu	TACATGACACTCCAGCCTTTG
CYP2D6	CYP2D6_For_L_Enzu	ACTCATCACCAACCTGTCATC
	CYP2D6_Rev_L_Enzu	CCTCCGGCTTCACAAAGT
CYP39A1	CYP39A1_For_L_Enzu	TGCAAGCTACGCTGGATATT
	CYP39A1_Rev_L_Enzu	AATGCAACAGGAACAGCATTAG
CYP4X1	CYP4X1_For_L_Enzu	AAGCATCTCCTGGATCCTTTAC
	CYP4X1_Rev_L_Enzu	CCAAGTGATAGAAGACCCATCC
DKK1	DKK1_For_qRT_C42B-Enzu	TTCTGTTTGTCTCCGGTCATC
	DKK1_Rev_qRT_C42B-Enzu	CTTGGTACACACTTGACCTTCT
ELOVL4	ELOVL4_For_L_Enzu	CCTGGTCCATCGCAGATAAG
	ELOVL4_Rev_L_Enzu	AGCCACACAAACAGGAGATAA
FAM92A1	FAM92A1-qRT-lnCap-Enzu_For	CTCACAGCAAGGAATCGAGAA
	FAM92A1-qRT-lnCap-Enzu_Rev	GCTGGGCAGTAACAACCTTA
FHL2	FHL2_For_L_Enzu	CCTGAACTGCTTCTGTGACTT
	FHL2_Rev_L_Enzu	CCGTTCCTCAAAGGAGATGTATT
GPAT2	GPAT2_For_qRT_C42B-Enzu	GTTCGGAGGCTCTGCTATTT
	GPAT2_Rev_qRT_C42B-Enzu	TCCCTGAGAGGAGGTTCTG
HBA2	HBA2_For_qRT_C42B-Enzu	GGACCCGGTCAACTTCAA
	HBA2_Rev_qRT_C42B-Enzu	CTCACAGAAGCCAGGAACTT
HIST1H1D	HIST1H1D-qRT-lnCap-Enzu_For	GGCAGTGGCAGCTTCTAA
	HIST1H1D-qRT-lnCap-Enzu_Rev	CTCTTGAGGCCAAGCTTGATA
HOXD10	HOXD10-qRT-lnCap-Enzu_For	CGAAGTGCAGGAGAAGGAAA
	HOXD10-qRT-lnCap-Enzu_Rev	CTTAGTGTAAGGGCACCTCTTC
HOXD11	HOXD11-qRT-lnCap-Enzu_For	AGCGCTGTCCCTATACCA
	HOXD11-qRT-lnCap-Enzu_Rev	AGGTTGAGCATCCGAGAGA
HOXD13	HOXD13-qRT-lnCap-Enzu_For	GAGGAAGAAGAGAGTGCCTTAC
	HOXD13-qRT-lnCap-Enzu_Rev	GTTCGTAGCAGCCGAGATAC
LBX1-AS1	LBX1-AS1-qRT-lnCap-Enzu_For	GGACTCCAGTCCCTCCTT
	LBX1-AS1-qRT-lnCap-Enzu_Rev	CCCACTCTCTCCCTCTGTAG

MFAP2	MFAP2_For_qRT_C42B-Enzu	AGATCGACAACCCAGACTACTA
	MFAP2_Rev_qRT_C42B-Enzu	GGATGACTTCCTGTTGGACTT
MIR4737	MIR4737_For_qRT_C42B-Enzu	TGCACAGGATGCGAGGAT
	MIR4737_Rev_qRT_C42B-Enzu	TGTGTGGCTGTGAGGCA
MT1G	MT1G _For_qRT_C42B-Enzu	GGAGCAGCAGCTCTTCTTG
	MT1G_Rev_qRT_C42B-Enzu	CTAGTCTCGCCTCGGGTT
PDPN	PDPN-qRT-lnCap-Enzu_For	CATCGAGGATCTGCCAACTT
	PDPN-qRT-lnCap-Enzu_Rev	TGTGTGTCTCCATCCACTTTC
PRTFDC1	PRTFDC1-qRT-lnCap-Enzu_For	AACGCTGGCTGGAAAGAA
	PRTFDC1-qRT-lnCap-Enzu_Rev	CCAACAAACTGGCTACCTTAATC
PTPRG-AS1	PTPRG-AS1-qRT-lnCap-Enzu_For	CATGCCATTCTCCTGCCTTA
	PTPRG-AS1-qRT-lnCap-Enzu_Rev	TCGAGACCATCCTGGCTAAT
RIBC2	RIBC2_For_qRT_C42B-Enzu	AGCAGCAAAGGGAATGGAA
	RIBC2_Rev_qRT_C42B-Enzu	CTTGGCTGTCTCGTCAAACT
SLC12A5	SLC12A5_For_L_Enzu	GTCGGTGGCAGAGAAGAATAAG
	SLC12A5_Rev_L_Enzu	CGTTGGACTGGTTCAAGTTCT
SLC24A4	SLC24A4_For_L_Enzu	GGTGATGGTGGACGAGATTATG
	SLC24A4_Rev_L_Enzu	GGTCCTGGGTCCAAACTTATT
SLC27A6	SLC27A6_For_L_Enzu	TGAGCAGGGTTGGTGTATTC
	SLC27A6_Rev_L_Enzu	TGCTTATAAGGCCCAGCATAG
SLC30A3	SLC30A3_For_L_Enzu	CCTTACGCTCACTTACCATGT
	SLC30A3_Rev_L_Enzu	GAGAATCCAAACCGGGAGTAG
SLC35D1	SLC35D1_For_L_Enzu	GGCAGAGAGCCTGTTTAGTT
	SLC35D1_Rev_L_Enzu	GAGGGTGAACTGCAGAAGAA
SLC47A1	SLC47A1_For_L_Enzu	ACCGTTTCCCTGCTGATTAC
	SLC47A1_Rev_L_Enzu	ATGATGTCTCGGTCGGTAGTA

Table S2. List of SLCs-genes differentially expressed in LNCaP and C4-2B-enzalutamide resistant cells.

LNCaP [Upregulated]	C4-2B [Upregulated]	LNCaP [Downregulated]	C4-2B [Downregulated]	LNCaP/C4-2B [Downregulated]	LNCaP/C4-2B [Upregulated]
SLC12A5	SLC16A8	SLC35A3	SLC3A2	SLC39A7	SLC10A3
SLC13A4	SLC17A6	SLC38A2	SLC39A3	SLC35E2B	SLC5A2
SLC19A3	SLC35F4	SLC35F5	SLC26A2	SLC25A37	SLC8A2
SLC1A2	SLC44A5	SLC25A1	SLC35A4	SLC25A36	SLC35D1
SLC1A3	SLC22A16	SLC39A9	SLC38A1	SLC22A18	SLC12A4
SLC1A5	SLC27A3	SLC7A2	SLC2A4RG	SLC16A14	SLC35F1
SLC20A1	SLC26A4	SLC25A23	SLC16A10	SLC25A5	SLC26A10
SLC22A15	SLC6A17	SLC52A2	SLC38A7	SLC29A2	SLC16A4
SLC23A2	SLC40A1	SLC6A16	SLC19A1	SLC31A1	SLC4A8
SLC23A3	SLC35A1	SLC22A23	SLC38A9	SLC7A11	SLC47A1
SLC24A4	SLC4A10	SLC35E2	SLC30A7	SLC25A45	SLC1A4
SLC25A13	SLC11A1	SLC37A4	SLC35E4	SLC27A5	SLC18B1
SLC25A14	SLC13A3	SLC46A1	SLC9A6	SLC41A3	SLC17A7
SLC25A17	SLC44A3	SLC25A21-AS1	SLC37A1	SLC12A7	SLC2A10
SLC25A18	SLC39A1	SLC6A9	SLC9A7	SLC50A1	SLC25A28
SLC25A19	SLC31A2	SLC38A4	SLC7A1	SLC35F2	SLC25A46
SLC25A24	SLC16A2	SLC2A12	SLC16A6	SLC39A14	SLC4A11
SLC25A27	SLC39A6	SLC6A20	SLC16A13	SLC12A8	SLC29A1
SLC25A32	SLC44A2	SLC16A7	SLC36A4	SLC39A4	SLC41A2
SLC25A43	SLC35C2	SLC45A1	SLC37A2	SLC35E1	SLC4A2
SLC25A53	SLC39A10	SLC6A3	SLC16A9	SLC2A4	SLC25A20
SLC25A5P4	SLC44A1		SLC2A6	SLC33A1	SLC17A5
SLC25A6P5	SLC10A5		SLC22A3	SLC10A7	
SLC26A3			SLC22A1	SLC25A25	
SLC27A4				SLC22A17	
SLC27A6				SLC4A4	
SLC2A13				SLC9A3R2	
SLC2A3				SLC35C1	
SLC2A8				SLC2A1	
SLC30A1				SLC27A1	
SLC30A3				SLC25A33	
SLC30A6				SLC25A10	
SLC35A5				SLC43A2	
SLC35B3				SLC41A1	
SLC35F6				SLC51A	
SLC35G1				SLC44A4	
SLC37A3				SLC6A8	
SLC38A3				SLC16A3	
SLC4A5				SLC4A7	
SLC5A12				SLC45A3	
SLC5A5				SLC4A9	
SLC5A6				SLC34A3	
SLC6A15				SLC25A48	
SLC7A5P1				SLC6A11	
SLC8A1				SLC26A1	
				SLC9A3R1	
				SLC43A1	
				SLC26A4-AS1	
				SLC15A2	
				SLC30A4	
				SLC9A2	
				SLC7A8	
				SLC22A31	
				SLC23A1	
